# The Role of Positron Emission Tomography Imaging in Primary Bone Tumours: A Narrative Review

**DOI:** 10.3390/jcm14082624

**Published:** 2025-04-11

**Authors:** Shihabul Hassan, Rishabh Suvarna, Hasaam Uldin, Mohsin Hussein, Rajesh Botchu

**Affiliations:** 1 Department of General Medicine, Buckinghamshire Healthcare NHS Trust, Aylesbury HP21 8AL, UK; shihabul.hassan1@nhs.net; 2 School of Medicine, Worsely Building, University of Leeds, Leeds LS2 9JT, UK; rishabhrsuvarna@gmail.com; 3 Department of Musculoskeletal Radiology, Royal Orthopaedic Hospital NHS Foundation Trust, Birmingham B31 2AP, UK; hasaam.uldin1@nhs.net; 4 Department of Radiology, University Hospitals of Leicester, Leicester LE2 7LX, UK; mohsin.hussein@nhs.net

**Keywords:** sarcoma, Ewing, positron emission tomography computed tomography, fluorodeoxyglucose F18, osteosarcoma, bone neoplasms, chondrosarcoma

## Abstract

Primary malignant bone tumours can pose significant diagnostic and therapeutic challenges due to inter-tumour heterogeneity. While traditional imaging modalities such as radiography, MRI (magnetic resonance imaging), and CT (computed tomography) remain essential for initial evaluation and staging, emerging evidence underscores the evolving role of positron emission tomography (PET), particularly PET/CT with Fluorodeoxyglucose ([18F] FDG), in the comprehensive management of bone sarcomas. This narrative review aims to critically summarise the available literature on PET imaging’s utility in the management of primary bone tumours including osteosarcoma, chondrosarcoma, and Ewing sarcoma. Despite limitations like inconsistencies in standard uptake value (SUV) cutoffs and reduced pulmonary resolution, PET/CT is valuable for staging, assessing response to neoadjuvant chemotherapy, predicting histological outcomes, detecting recurrence, and guiding biopsy in metabolically active tumour sites. Further large-scale, prospective studies are warranted to standardise protocols and establish PET’s definitive role in sarcoma management.

## 1. Introduction

Primary malignant bone tumours represent a rare and heterogenous group of tumours, accounting for approximately 0.2% of all cancers, with an annual incidence of about 8.5 cases per million people in Europe [1,2]. Current national guidelines for the diagnosis and management of primary bone tumours in the United Kingdom (UK) emphasize a structured approach comprising specific imaging and biopsy protocols before surgery.

For suspected bone tumours, a radiograph is typically the first investigation, with specific features suggestive of malignancy including bone destruction, new bone formation, aggressive periosteal reaction, and soft tissue swelling. However, a “normal” radiograph does not rule out a bone sarcoma, and persistent pain, particularly night pain, warrants further investigation with urgent referral for magnetic resonance imaging (MRI) [3]. In patients under 40 years of age, radiographs (in two planes) and basic blood tests are typically performed before referral. Further imaging, such as MRI or computerised tomography (CT), is required for local and distant staging. In patients over 40, there is a higher likelihood of metastatic disease, so more extensive pre-referral investigations are advised, including CT of the chest, abdomen, and pelvis; an isotope bone scintigraphy; and a myeloma screen. If the lesion is solitary, referral to a regional sarcoma centre for a potential primary malignant bone tumour is recommended. While imaging techniques are crucial in the diagnosis and staging of bone tumours, biopsy remains the mainstay for confirming the tumour’s histology and guiding treatment decisions [3]. 

Positron emission tomography (PET) or PET with CT (PET-CT) is not currently considered standard care in the initial diagnosis of bone sarcomas. However, PET-CT, particularly with the radiotracer Fluorodeoxyglucose ([18F] FDG), is increasingly used for staging and monitoring treatment response [3]. The aim of this article is to critically summarise the available literature on the utility of PET scanning in both the diagnosis and management of bone cancers and illustrate examples of its usage from our experience. It explores the role of PET in detecting primary bone tumours, staging disease, and monitoring treatment response while also comparing its performance to other imaging techniques such as CT, MRI, and bone scintigraphy.

A literature search was performed using electronic databases, including PubMed, Scopus, and Web of Science. The search strategy incorporated a combination of medical subject headings (MeSHs) and free-text terms related to “sarcoma”, “PET imaging”, “[18F] FDG-PET”, “diagnosis”, “staging”, “treatment response”, and “prognosis”. Boolean operators (AND/OR) were utilised to refine the search results, and additional relevant articles were identified through a manual screening of the reference lists of included studies.

Studies were selected based on predefined inclusion and exclusion criteria. Eligible studies included meta-analyses and systematic reviews published in peer-reviewed journals that focused on PET imaging in sarcoma, including various tracers such as [18F] FDG 68Ga-labeled radiotracers. Articles published in English from 2005 were included. Studies were excluded if they were case reports, editorials, or conference abstracts; focused on other imaging modalities without a specific analysis of PET imaging; or investigated non-sarcoma malignancies or bony metastasis from non-sarcoma primary cancers.

## 2. Brief History of PET Imaging

The concept of PET imaging began in 1951 with early work by Professor William H. Sweet at Massachusetts General Hospital (MGH) and Wrenn FR Junior at Duke University, who first used nuclear disintegration and coincidence detection of positrons to detect brain tumours [4]. During the 1960s, Kuhl and Edwards improved this technology by developing back projection and imaging reconstruction techniques for single-photon tomography, which could be overlaid onto CT images. However, output quality was often blurred due to high levels of noise [4]. By the 1970s, significant advances were made, including the development of the first PET tomograph by Michael E. Phelps in 1973. This early PET technology was groundbreaking in its ability to visualise metabolic activity and tissue function, a crucial aspect in oncology. 

For many years, PET was performed independently of high-resolution anatomic imaging such as CT. Before 2001, the fusion of PET and CT images was achieved using software-based techniques, and its clinical applicability was mainly limited to brain imaging [1]. However, since 2001, the advent of commercially available combined PET/CT scanners allowed for the simultaneous acquisition of both functional PET and anatomic CT images. This integration provided a more accurate and comprehensive view of the tumour’s location, size, and metabolic activity.

## 3. Basic Science Behind PET Imaging

Positron emission tomography (PET) scans utilise radiopharmaceuticals produced by labelling suitable molecules with positron-emitting radionuclides. These latter undergo beta-positive decay to achieve nuclear stability by converting excess protons to neutrons [5]. As positrons are produced and travel a short distance (<1 mm), the encounter electrons found in biological tissue and subsequently undergo annihilation, losing energy and producing gamma rays emitted in nearly opposite directions. This can be detected through a phenomenon known as coincidence, involving multiple detectors simultaneously measuring this emission within a small time interval [5]. Thus, the total number of coincidence events is regarded to be proportional to the quantity of radiotracer present within the tissue, following adjustments for confounders such as photon attenuation and scattering.

As radiopharmaceuticals are administered to patients prior to the scan, tomographic PET scanners produce lines of responses (LORs) from each pair of coincident gamma ray photons emitted from the annihilation of positrons produced by radionuclides. Millions of LORs are collated as projection data in a sinogram, representing the possible trajectories of photons and revealing the location of the annihilation event. Various reconstructive algorithms can process these data. The most common and convenient method is filtered back projection, involving an evenly distributed projection of the detected counts across the respective LOR, with contributions per LOR superimposed across the full image space to generate estimates of the original radiotracer distribution. However, this technique often results in blurry output by assuming equal distribution of activity along each LOR, requiring an additional ramp filter with low-pass adjustments like Hann, Shepp-Logan filters to adjust the weight of different frequencies and remove noise. Iterative processes can be used to overcome these limitations, enhancing accuracy but requiring significant computational effort, by modelling statistical noise and accounting for physical perturbations, such as Maximum-Likelihood Expectation-Maximisation and Ordered Subsets Expectation-Maximisation.

This enables an activity distribution relative to angular correlations of the emissions and axial position, which is then subsequently converted into a distribution of radioactivity. PET imaging, thus, highlights both structural and functional abnormalities of imaged tissue, gaining utility and popularity within oncology [6]. A key radiotracer used is Fluorodeoxyglucose [18F] FDG, which allows for the assessment of tumour metabolism. [18F] FDG is transported into cells and phosphorylated by hexokinase, becoming trapped within the cell as [18F] FDG-6-phosphate. This process correlates with the rate of glycolysis in tissues, allowing for quantitative metabolic imaging. The standard uptake value (SUV), including both average and maximum SUVs, is used to quantify tumour metabolism [7].

## 4. Application to Primary Bone Tumours

In addition to traditional, well-established anatomic imaging techniques, metabolic PET imaging has become a key area of sarcoma research. It has emerged as a useful tool in determining metabolic rates of primary bone tumours, monitoring clinical and histological tumour responses to neoadjuvant therapy, distinguishing viable sarcoma tissue from changes caused by treatment, and determining prognosis post-therapy.

### 4.1. Osteosarcoma

Osteosarcoma is the most common primary malignant bone tumour in children and young adults, typically affecting patients in their second and third decades of life. Due to recent advancements in chemotherapy, the development of rigorous classification systems to guide treatment, and the aggressive nature of advanced-stage osteosarcoma, there is an increasing need for more accurate tumour staging pre- and post-treatment for patients with osteosarcoma. A review of PET/CT imaging by Oh et al. revealed that its sensitivity in detecting osteosarcoma ranged from 95% to 98%, with specificity ranging between 77% and 97%, significantly outperforming radiographs (sensitivity 83%, specificity 78%) [8,9]. PET/CT was also found to be more accurate than CT alone or dedicated MRI for detecting bone metastases [10]. This is routinely combined with a high-resolution CT of the chest to assess for pulmonary nodule and metastasis [11,12]. Another recent meta-analysis assessed the role of PET/CT in diagnosing and staging osteosarcoma, revealing that PET/CT imaging had 100% sensitivity for detecting primary lesions in 14 out of 26 studies. For lung metastases, the pooled sensitivity was 81% (95% Confidence Interval (CI): 72–88%), and the specificity was 94% (95% CI: 89–97%) [13]. In the detection of bone metastases, six studies showed a pooled sensitivity of 93% (95% CI: 87–97%) and specificity of 97% (95% CI: 96–98%) [10,13].

**Figure 1:**
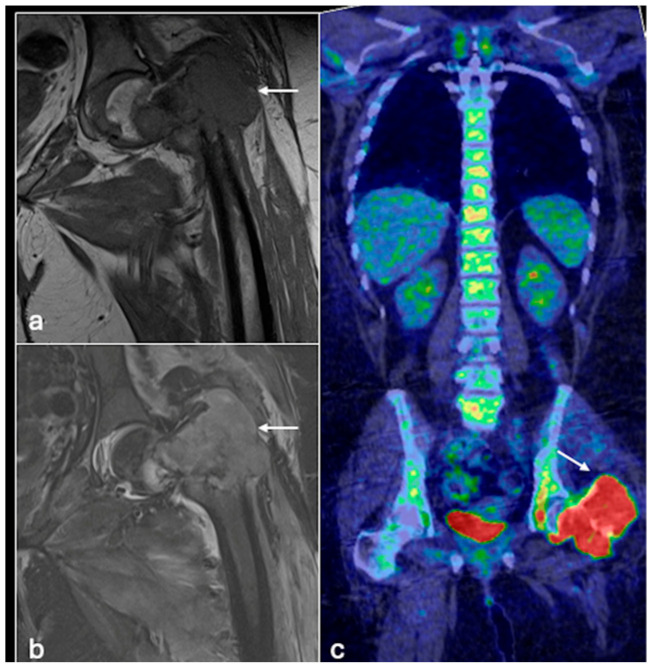
Coronal T1 (**a**), STIR (Short Tau Inversion Recovery) (**b**), MRI images and PET-CT (**c**) showing a tumour (osteosarcoma) involving the left proximal femur with marked increased uptake within the tumour on PET (arrows).

**Figure 2:**
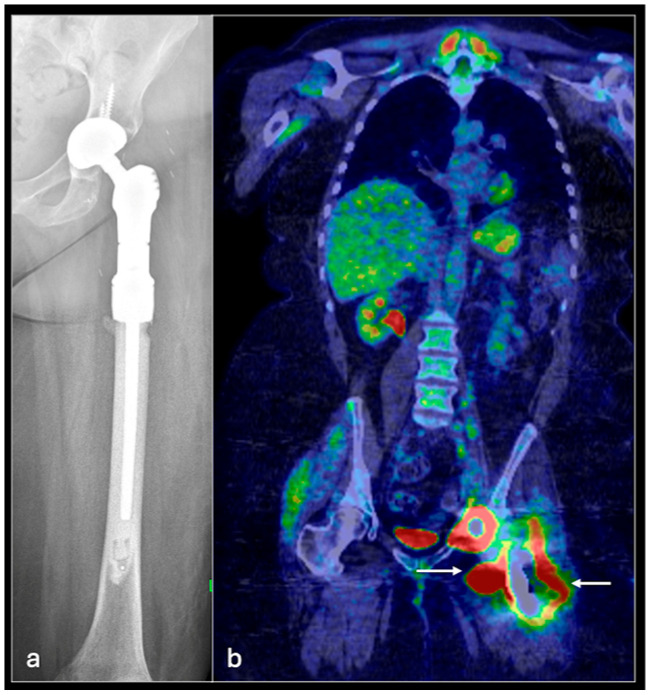
AP (Anteroposterior) radiograph of the left femur (**a**) with proximal femoral EPR (endoprosthetic replacement). PET/CT (**b**) coronal showing an extensive local recurrence (arrows) of osteosarcoma around the left proximal femoral EPR.

These findings demonstrate the value of PET/CT in differentiating primary bone lesions and in staging both bone and lung metastases [Figure 1 and Figure 2]. PET/MRI performs similarly to PET/CT but is better able to visualise soft tissue pathology and foregoes ionising radiation, making it a suitable choice of imaging, particularly for the paediatric population who require long-term follow-up [14]. However, it is limited by the poor resolution of pulmonary nodules, often requiring follow-up CT chest scans.

[18F] FDG PET/CT and PET/MRI also show promise in identifying chemotherapy-resistant patients with osteosarcoma, effectively predicting histological early response to chemotherapy and risk-stratifying those likely to experience local recurrence. The traditional assessment relies on the histological evaluation of tumour necrosis in resected specimens, which, while prognostic, is only available post-surgery. [18F] FDG PET/CT offers a non-invasive, early surrogate marker for histological necrosis, potentially accelerating the assessment of NACT (neoadjuvant chemotherapy) efficacy. Changes in SUVmax following NACT show promise in predicting tumour response. High metabolic activity, as measured by [18F] FDG uptake in chemotherapy-naive tumours and persistently elevated uptake post-chemotherapy, is associated with worse survival, suggesting that [18F] FDG PET accurately reflects osteosarcoma response to chemotherapy.

A recent systematic review demonstrated a strong association between decreases in SUVmax and Recurrence Free Survival for osteosarcomas with Level II–III evidence, presenting a strong case for its use in prognostication [15]. In a network meta-analysis and regression of 20 studies, 858 patients with osteosarcoma specifically confirmed the above, showing that SUVmax, metabolic tumour volume, and total lesion glycolysis rates were associated with survival, showing potential in stratifying patients at risk of worse outcomes [16].

A meta-analysis examined the utility of [18F] FDG PET in assessing histological response to neoadjuvant chemotherapy in osteosarcoma patients [17]. The study, which included eight articles encompassing 178 patients, evaluated two main criteria: SUV after chemotherapy (SUV2) ≤ 2.5 and the ratio of SUV after to before chemotherapy (SUV2:1) ≤ 0.5 [17]. The results demonstrated that SUV2 ≤ 2.5 had a sensitivity of 0.734 and specificity of 0.864, while SUV2:1 ≤ 0.5 showed a sensitivity of 0.690 and specificity of 0.653. The area under the Summary Receiver Operating Curve (SROC) curve was 0.81 for SUV2 ≤ 2.5 and 0.72 for SUV2:1 ≤ 0.5, indicating excellent and acceptable discrimination, respectively. Likelihood ratios further supported the superiority of SUV2 ≤ 2.5, with a positive likelihood ratio of 5.397 compared to 1.989 for SUV2:1 ≤ 0.5. Negative likelihood ratios were 0.308 and 0.475, respectively. These findings suggest that [18F] FDG PET-CT parameters, particularly SUV2 ≤ 2.5, have predictive value for tumour response to neoadjuvant chemotherapy in osteosarcoma. However, the authors caution that negative results should be interpreted carefully, as they may not definitively rule out a good response to therapy [17].

In addition to SUVmax, Im et al. evaluated other key parameters including metabolic tumour volume (MTV), and total lesion glycolysis (TLG), both after a single course of chemotherapy and upon completion of treatment in patients with osteosarcoma [18]. The results revealed that MTV at an SUV threshold of 2 g/mL was strongly correlated with pre-therapy MRI tumour volumes (r = 0.91). After chemotherapy, significant differences in PET/CT indices were observed between good responders (≥90% necrosis) and poor responders (<90% necrosis), with post-therapy SUVmax, MTV, and TLG values—as well as their ratios relative to the baseline—showing strong associations with histological necrosis fractions (*p* < 0.05). Notably, PET/CT measurements taken after just one cycle of chemotherapy could already distinguish responders from non-responders, with interim SUVmax, MTV, and TLG demonstrating high sensitivity and specificity in predicting treatment outcomes. Post-therapy SUVmax values below 3 g/mL had 100% sensitivity and 88.9% specificity for identifying good responders, while combined metabolic and volumetric indices like MTV and TLG comprehensively captured tumour burden, especially in tumours with heterogeneous [18F] FDG uptake. In contrast, MRI measurements were less effective at distinguishing treatment responses, likely because MRI captures structural rather than functional changes in tumours [18].

These findings highlight the potential of PET/CT as a powerful, non-invasive tool for early response assessments in osteosarcoma, which could inform timely treatment adjustments and optimize patient outcomes. However, the study’s small sample size (*n* = 20) and the use of diverse chemotherapy regimens limit the generalizability of the results. Larger, standardised studies are needed to validate these findings and establish clear clinical guidelines for integrating PET/CT into osteosarcoma treatment protocols.

### 4.2. Chondrosarcoma

Chondrosarcomas are malignant cartilaginous neoplasms which present significant diagnostic challenge given their diverse histological features. The identification of chondrosarcomas (distinguishing them from benign growths) and accurate grading are both crucial in guiding treatment decisions.

**Figure 3:**
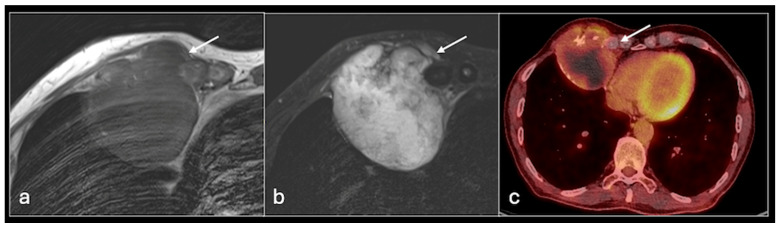
Axial T1 (**a**), STIR (**b**), MRI images and PET-CT (**c**) showing large chondrosarcoma in relation to right chest wall with mild increased uptake on PET-CT (arrow).

**Figure 4:**
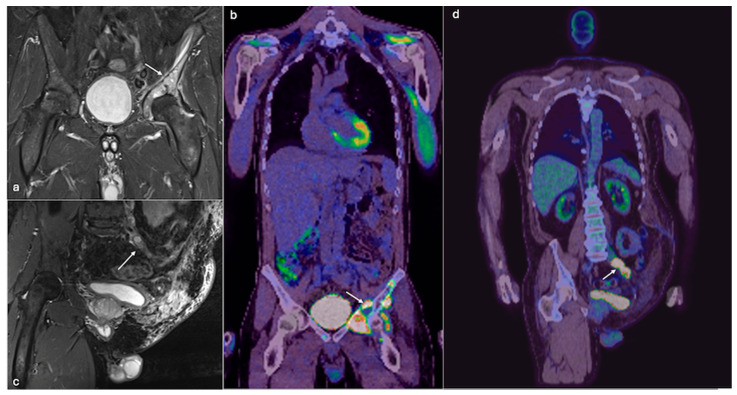
Coronal STIR MRI images (**a**) showing a large chondrosarcoma involving the right hemipelvis (arrow) with increased uptake on coronal PET/CT (**b**), treated with left hindquarter amputation (**c**). PET/CT coronal (**d**) showing recurrence with left common iliac vein tumour thrombus showing increased uptake (arrow). (STIR—Short Tau Inversion Recovery).

Benign cartilage lesions, such as enchondromas, typically receive a “watch and wait” approach, with local intervention, such as marginal excision, reserved for symptomatic cases. In contrast, high-grade chondrosarcomas necessitate early surgery due to their malignant nature and potential for aggressive behaviour, while patients with de-differentiated chondrosarcoma further benefit from neo-adjuvant chemotherapy prior to surgery or palliative radiotherapy in cases of unresectable lesions [19]. Biopsy alone is often insufficient for establishing a definitive diagnosis or determining the histological grade in cartilaginous growths and can underestimate the degree of differentiation [20]. Anatomical imaging by itself is also insufficient to accurately differentiate malignancies from benign chondroid growths [21]. Functional imaging such as PET scanning, especially in combination with conventional anatomical imaging, has emerged as a useful tool in diagnosing, staging, and monitoring chondrosarcomas [Figure 3 and Figure 4].

A recent meta-analysis of six studies evaluating the diagnostic accuracy of [18F] FDG PET for chondrosarcoma revealed a pooled sensitivity of 84% (95% CI, 46% to 97%) and specificity of 82% (95% CI, 55% to 94%) [22]. The area under the curve (AUC) was found to be 0.89 (95% CI, 0.86 to 0.92), indicating good overall diagnostic performance [22]. However, there was substantial heterogeneity among the included studies, with I^2^ statistics of 86.90% for sensitivity and 70.32% for specificity. For PET/CT, the authors found an even greater pooled sensitivity of 94% (95% CI, 86% to 97%), specificity of 89% (95% CI, 82% to 93%), and area under the curve (AUC) of 0.92 (95% CI, 0.89 to 0.94) with low heterogeneity for both sensitivity (15.79%) and specificity (0%) among seven included studies [22].

The same study evaluated the utility of PET imaging in grading chondrosarcomas, concluding that [18F] FDG PET may be useful in distinguishing between low-grade (Grade 1) and high-grade (Grade 2/3) chondrosarcomas but may have limitations in differentiating between benign lesions and Grade 1 chondrosarcomas or between Grade 2 and Grade 3 chondrosarcomas [22]. The authors theorised that highly differentiated chondrosarcomas would have an increased uptake of [18F] FDG due to increased tumour metabolic activity and found this to be the case, with Grade 1 chondrosarcomas demonstrating significantly lower SUVmax values compared to Grade 2 (pooled weighted mean difference = −1.94; 95% CI, −2.76 to −1.12; *p* < 0.001) [22]. Another retrospective cohort study of 104 patients identified 100% specificity and 88.9% sensitivity when using an SUVmax cutoff of 13.3 alongside radiological parameters to diagnose dedifferentiated chondrosarcomas, holding prognostic value in being better able to risk-stratify and support these patients early on with more aggressive surgical excision, adjuvant therapy, treatment for metastases, and closer monitoring for recurrence. However, there remain inconsistencies across institutions on pre-defined SUVmax cut-offs to differentiate chondromas from chondrosarcomas or between chondrosarcomas of different grades. Whilst an SUVmax cutoff of 2.0–2.2 has been previously specified to differentiate between chondromas and chondrosarcomas, this remains poorly standardised across the literature, as systematic reviews have reported.

In addition to detecting, staging, and grading cartilaginous neoplasms, PET can identify regions of the highest metabolic activity (SUVmax) to guide biopsies, enabling the sampling of the most metabolically active region, potentially reducing false-negative results and re-biopsy rates [22]. Furthermore, in postoperative surveillance, changes in [18F] FDG avidity can indicate relapse or sarcomatous transformation, although further research is required in this regard [22]. Indeterminate nodules in chondrosarcoma are not uncommon; hence, high-resolution CT is required in addition to PET to identify these [23].

### 4.3. Ewing Sarcoma

Ewing sarcoma (ES), the second most common primary bone tumour, has seen significant improvements in patient outcomes over recent decades. With the advent of multimodal therapy combining chemotherapy, surgery, and radiotherapy, survival rates have improved to 60–70% [24]. Current data from the American Cancer Society indicates a 5-year relative survival rate of 63% for all Surveillance, Epidemiology, and End Results (SEER) stages combined, with rates exceeding 70% for localized disease [25]. However, 30–40% of patients still develop local and/or distant recurrent disease, typically between 2 and 10 years after diagnosis, and the presence of metastatic disease remains the single greatest adverse prognostic factor in paediatric patients with ES, underscoring the need for accurate diagnostic and monitoring tools [26]. Like osteosarcoma, traditional bone marrow aspiration and biopsy is prone to sampling errors due to intratumour heterogeneity. A contemporary multicentre phase II study prospectively enrolled 45 patients with extra-pulmonary metastases, comparing PET/CT versus bone marrow aspiration and biopsy for staging [27]. A central analysis showed that PET/CT outperformed bone marrow biopsy in detecting bone marrow involvement with diffuse [18F] FDG uptake, accurately capturing age-related changes in bone marrow, and identified further bone metastases with focal [18F] FDG uptake and cortical destruction undetected by biopsy [27].

**Figure 5:**
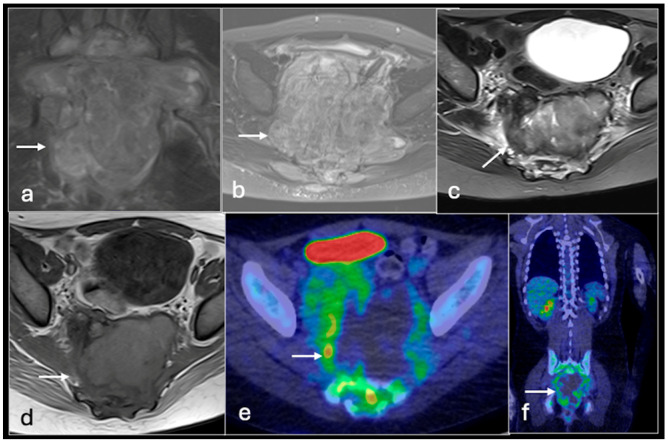
Coronal STIR (**a**), axial STIR (**b**), T2FS (T2 fat suppressed) (**c**), T1 (**d**), MRI images and PET/CT (**e**), and coronal PET/CT (**f**) showing large sacral Ewing sarcoma (arrow) (**a**,**b**) with a marked decrease following adjuvant treatment (**c**,**d**) and mild uptake in the periphery of the lesion and no central uptake (arrow) (**e**,**f**) (STIR—Short Tau Inversion Recovery).

**Figure 6:**
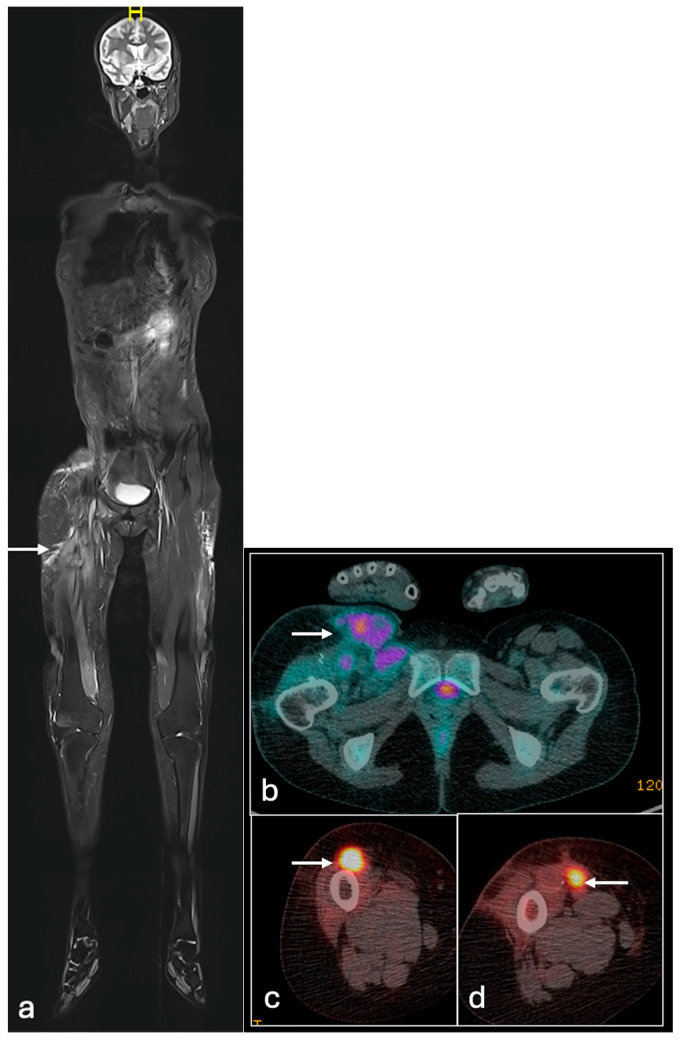
Whole-body MRI (**a**) showing ES recurrence in the right thigh (arrow); axial PET/CT (**b**–**d**) showing a multifocal recurrence of a tumour in the right groin and thigh (arrows).

A recent meta-analysis comprising 13 trials involving 689 lesions reported a pooled sensitivity of 86% (95% CI: 82–89%) and specificity of 80% (95% CI: 75–85%) for [18F] FDG PET and PET/CT in detecting ES [28]. The diagnostic odds ratio (DOR) of 29.22 (95% CI: 16.49–51.78) and area under the curve (AUC) of 0.9147 further support its high diagnostic performance [28]. Another study in the paediatric population evaluated the utility of [18F] FDG PET/CT in detecting and staging ES compared to conventional imaging modalities like CT and MRI. The findings demonstrated that PET/CT has high sensitivity (92.6%) and moderate specificity (74.1%) for detecting ES lesions, with superiority in characterizing skeletal lesions compared to conventional imaging [29] [Figure 5 and Figure 6]. At initial staging, PET/CT provided diagnostic benefit in 27.8% of cases, detecting more true-positive lesions and reducing false-positives compared to CT. For bone metastases, PET/CT exhibited high sensitivity (83.9%) and specificity (93.2%), outperforming CT and scintigraphy in follow-up evaluations for both ES and osteosarcoma [29].

PET/CT performed similarly to conventional imaging in detecting lymph node metastases, although some false-positives were noted during follow-up, attributed to reactive changes. In recurrence detection, PET/CT demonstrated high sensitivity (89.9%) and specificity (92.6%). The ability of PET/CT to provide whole-body screening in a single examination proved advantageous for comprehensive staging. However, the study highlighted a significant limitation of PET/CT in detecting pulmonary metastases [29,30]. PET/CT showed a lower sensitivity compared to dedicated chest CT for small lung nodules. This was attributed to the lower resolution of the CT component in PET/CT, especially during shallow breathing, and the partial volume effect from respiratory motion during PET acquisition, which reduced the recorded activity in small lung lesions [29]. False-negative results were particularly noted for lung nodules smaller than 7.5 mm. In conclusion, while [18F] FDG PET/CT demonstrates utility in the diagnosis, staging, and recurrence monitoring of ES, especially for skeletal lesions, authors recommended that it should be used with dedicated chest CT for the optimal detection of pulmonary metastases [29,30].

Huang et al. further proposed that SUVmax measured by pretreatment [18F] FDG PET/CT can predict overall survival, demonstrating its utility in assessing the primary tumour’s prognosis. The study found that patients with an SUVmax > 5.8 had significantly lower survival times (1236 days compared to 627 days for those with an SUVmax < 5.8), and that a higher SUVmax was related to poorer prognosis [25]. This threshold provided a quantitative benchmark for risk stratification. Furthermore, a multivariate analysis revealed that SUVmax was independently associated with overall survival, with a hazard ratio of 42.9 (CI, 3.63–508.26; *p* = 0.003), indicating a substantially increased risk of mortality with higher SUVmax values. Though the study supports that SUVmax using [18F] FDG PET/CT demonstrates independent properties for the prediction of survival, it was a single-centre retrospective analysis with only 34 patients, potentially limiting the generalizability of the findings [25]. Table 1 depicts the comparative abilities of [18] F-FDG PET/CT, 99m Tc-labelled methylene diphosphonate (^99m^Tc-MDP) Skeletal Scintigraphy, and whole-body MRI to detect skeletal metastases in patients with primary bone tumours, as published in two recent systematic reviews [31,32].

## 5. Limitations and Future Research Directions

Despite its utility in detecting, staging, re-staging, prognosticating, and monitoring the treatment response of primary bone tumours, PET imaging is not without limitations. For the initial workup of bone lesions, conventional imaging modalities like plain radiographs, MRI, and high-resolution CT remain the first-line options due to their practicality and wider availability compared to PET, with CT proving superior for detecting lung metastases, particularly in patients with chondrosarcoma.

PET is also limited in primary staging, except perhaps in paediatric cases where it could help detect intraosseous skip metastases in ambiguous MRI findings, though this remains speculative without supporting data [7]. Quantitative PET parameters like SUV provide non-invasive surrogates for tumour grading but cannot reliably distinguish between intermediate and high-grade tumours due to overlapping SUV ranges, reinforcing the necessity of biopsy for definitive diagnosis [7,35]. Tumour heterogeneity further complicates PET interpretation, as [18F] FDG uptake varies within different tumour regions, reflecting varying metabolic activity and areas of necrosis. High-grade sarcomas may reach peak [18F] FDG activity hours after injection, while benign or low-grade lesions peak much earlier; single-timepoint SUVs may miss this dynamic tumour metabolism. Although cutoff SUV values have been proposed for malignancy detection, these thresholds have inconsistent sensitivity and specificity, often failing to differentiate aggressive benign lesions from malignant bone tumours.

FAP (fibroblast activation protein) ligand inhibitor (68Ga-FAPI) PET/CT has shown promise in the diagnosis and management of soft tissue sarcomas, as these tumour cells express FAP. 68Ga-FAPI has been shown to demonstrate increased uptake in both primary tumour and metastasis with a relatively low background signal. This may be particularly useful for liposarcoma and high-grade soft tissue sarcomas [33,36,37].

Ultimately, while PET shows promise in guiding biopsies, predicting chemotherapy response, and monitoring for recurrence, the small sample sizes used in current studies and the lack of standardised protocols prevent definitive clinical recommendations. Larger, prospective studies are essential to refine PET’s role, optimize SUV thresholds, and establish clear indications for its use across the sarcoma care continuum.

## Figures and Tables

**Table 1:** Comparative abilities of [18F]-FDG PET/CT, (^99m^Tc-MDP) Skeletal Scintigraphy, and whole-body MRI to detect skeletal metastases in patients with osteosarcoma, Ewing’s sarcoma, and chondrosarcoma.

	Osteosarcoma	Ewing’s Sarcoma	Chondrosarcoma
**BONE SCINITGRAPHY**			
Sensitivity	67% [31]	50% [31]	100% [33]
Specificity	78% [31]	95% [31]	94.7% [33]
Positive predictive value	50% [31]	80% [31]	45.5% [33]
Negative predictive value	88% [31]	84% [31]	100% [33]
**PET-CT**			
Sensitivity	100% [31]	88% [31]	100% [33]
Specificity	100% [31]	100% [31]	88.5% [33]
Positive predictive value	100% [31]	100% [31]	50% [33]
Negative predictive value	100% [31]	96% [31]	100% [33]
**WHOLE-BODY MRI**			
Sensitivity	83% [31]	88% [31]	100% *
Specificity	94% [31]	95% [31]	94% *
Positive predictive value	83% [31]	88% [31]	25% *
Negative predictive value	94% [31]	95% [31]	100% *

* These values are not exclusive to chondrosarcoma, as no study was found to have specifically investigated the role of whole-body MRI in its assessment [34].

## Abbreviations

STIR	Short tau Inversion Recovery
T2FS	T2 fat suppressed
EPR	Endoprosthetic replacement
FDG	Fluorodeoxyglucose
CT	Computed tomography
MRI	magnetic resonance imaging
PET	Positron emission tomography
SUV	Standard Uptake Value
MTV	Metabolic tumour volume
TLG	Total lesion glycolysis
ES	Ewing sarcoma
AUC	Area under the curve
CI	Confidence Interval
DMSA	Dimercaptosuccinic Acid
MDP	Methyl Diphosphonate
SUVmax	SUV maximum
